# Papel da Interleucina 18 e da Proteína Precursora do Trombo na Doença Arterial Coronariana

**DOI:** 10.36660/abc.20190176

**Published:** 2020-05-12

**Authors:** Carlos Scherr, Denilson Campos de Albuquerque, Roberto Pozzan, Kezia Ataide, Talita Ludmila, Fernanda Blanco, Claudio Martins Mangia

**Affiliations:** 1 Ministério da Saúde Brasília DF Brasil Ministério da Saúde – Cardiologia, Brasília, DF - Brasil; 2 Universidade do Estado do Rio de Janeiro Rio de Janeiro RJ Brasil Universidade do Estado do Rio de Janeiro – Cardiologia, Rio de Janeiro, RJ - Brasil; 3 Universidade do Estado do Rio de Janeiro Rio de Janeiro RJ Brasil Universidade do Estado do Rio de Janeiro – Cardiologia/Hipertensão, Rio de Janeiro, RJ - Brasil; 4 Hospital Universitário Pedro Ernesto Rio de Janeiro RJ Brasil Hospital Universitário Pedro Ernesto – Cardiologia, Rio de Janeiro, RJ - Brasil; 5 Fundação Pró Coração Rio de Janeiro RJ Brasil Fundação Pró Coração – Cardiologia, Rio de Janeiro, RJ – Brasil

**Keywords:** Doenças Cardiovasculares/mortalidade, Doença da Artéria Coronariana, Interleucina 18, Biomarcadores, Aterosclerose/tratamento Farmacológico, Síndrome Coronariana Aguda/ prevenção e controle

## Abstract

**Fundamento:**

A insuficiência coronariana constitui a principal causa de morte no mundo, e a identificação de pacientes de maior risco para doença arterial coronariana (DAC) constitui um desafio.

**Objetivos:**

Testar os biomarcadores interleucina 18 (IL-18) e proteína precursora do trombo (TpP), envolvidos na aterogênese, para auxílio na avaliação precoce de DAC.

**Métodos:**

Coorte transversal de 119 pacientes, estratificados em três grupos: Grupo I – síndrome coronariana aguda (39); Grupo II – DAC crônica (40); e Grupo III – controle, sem lesão coronariana, mas podendo apresentar fatores de risco para DAC (40). Análise estatística através do programa estatístico SPSS (Statistical Package for the Social Sciences) para Windows versão 17.0 de 2008. Fixou-se em 0,05 ou 5% (p<0,05) nível de significância e intervalo de confiança de 95%. Teste do qui-quadrado (χ2). Análise de variância (ANOVA), Teste de Tukey.

**Resultados:**

Idade média, 60,36±9,64 anos; prevalência do sexo feminino no Grupo III (65,0% p=0,002), porém sem significado estatístico para os valores médios de IL-18 e TpP. Os valores médios de IL-18 e TpP mostravam-se aumentados no Grupo I, quando comparados aos valores dos demais grupos: IL-18 = 1325,44±1860,13 ng/dL, p=0,002; TpP = 35,86±28,36 µg/mL, p<0,001. Na comparação dois a dois, observou-se que o Grupo I apresentou valor médio de IL-18 e TpP maior que o do Grupo II (IL-18 = 353,81±273,65 ng/dL; TpP = 25,66±12,17 µg/mL) e o do Grupo III (IL-18 = 633,25±993,93 ng/dL; TpP=18,00±8,45 µg/mL).

**Conclusão:**

Na vigência de DAC aguda, houve elevação desses biomarcadores, sugerindo relação com o processo de instabilidade da placa aterosclerótica, mas não com a fase crônica. (Arq Bras Cardiol. 2020; 114(4):692-698)

## Introdução

A doença arterial coronariana é, no século XXI, uma das principais manifestações de doença cardiovascular, e sua importância está em suas altas morbidade e mortalidade. A identificação dos pacientes de maior risco torna-se necessária para contribuir para melhora desse quadro e racionalização de custos.^1^ A medicina moderna tem evoluído rapidamente no âmbito da prevenção, da detecção precoce e do rastreamento de doenças, não se restringindo ao tratamento. Nesse sentido, a detecção precoce, associada ao tratamento imediato da doença cardiovascular (DCV), tornou-se um dos empreendimentos mais desafiadores para médicos e pesquisadores em todo o mundo. Numerosos biomarcadores têm sido estudados nos últimos anos no diagnóstico, no prognóstico, na predição de eventos adversos e no monitoramento terapêutico.^2^ No entanto, a primeira iniciativa em direção à prevenção é aplicar estratégias para identificar o indivíduo com probabilidade de apresentar eventos ateroscleróticos. Nesse sentido, além de os fatores de risco amplamente conhecidos contribuírem no processo de identificação de vulneráveis, o uso de biomarcadores envolvidos na aterogênese pode constituir peça-chave na avaliação do risco de doença arterial coronariana (DAC).^3 , 4^

A aterosclerose é uma doença inflamatória crônica, multifatorial, lenta e progressiva, resultante de inúmeras respostas celulares e moleculares específicas que levam à agressão endotelial, acometendo principalmente a camada íntima de artérias de médio e grosso calibre.

Em geral, a ruptura da capsula fibrosa da placa aterosclerótica leva a trombose, que, em menor proporção, pode resultar da erosão endotelial superficial. As placas que se rompem geralmente estão associadas a inflamação da íntima e da adventícia, hemorragia intraplaca, exposição de material trombogênico na circulação sanguínea disparando acúmulo de plaquetas, ativação da cascata de coagulação e deposição de fibrina.^5^

A resposta inflamatória na aterogênese consiste em mudanças funcionais em células endoteliais, linfócitos T, macrófagos derivados de monócitos, células dos músculos lisos e nos estágios iniciais, provocada pelo acumulo de lipídeos nas paredes das artérias.^6^ A ativação dessas células desencadeia a formação e interação de várias citocinas, moléculas de adesão, fatores de crescimento, acúmulo de lipídeos e proliferação de células do músculo liso. Além desses fatores, a resposta inflamatória pode ser induzida por estresse oxidativo (oxidação de lipoproteína de baixa densidade - LDL).^7 , 8^

Atualmente, novos biomarcadores vêm sendo estudados, em associação a síndrome coronariana aguda ou crônica e correlacionados ao valor prognóstico; este é o objetivo do presente estudo.

Neste estudo, foram avaliados dois biomarcadores: a proteína precursora do trombo (TpP) e a interleucina 18 (IL-18) em indivíduos com infarto agudo do miocárdio (IAM) com e sem supradesnivelamento de ST, angina instável (AI), doença arterial coronariana crônica e indivíduos que não apresentavam evidência de doença aterosclerótica coronariana obstrutiva, porém na presença de fatores de risco para DAC. A escolha dos biomarcadores foi baseada no envolvimento destes com o processo inflamatório (IL-18) e trombótico (TpP), inserido no contexto de aterosclerose e agudização da doença aterosclerótica. TpP é um biomarcador usado para estimativa de polímeros de fibrina solúveis. Níveis elevados de TpP são indicativos de estado pró-trombótico e de trombogênese em atividade. Nos pacientes com SCA, o aumento dos níveis desse biomarcador estão associados a maior risco de morte e complicações isquêmicas; portanto, a TpP tem-se mostrado um marcador independente para desfechos cardiovasculares adversos.^9^ O biomarcador IL-18 é uma citocina pró-inflamatória, intimamente relacionada com a instabilidade da placa aterosclerótica, sendo por isso boa preditora de eventos indesejáveis na SCA.^10 , 11^ Em estudo observacional, a IL-18 também se mostrou boa preditora de eventos cardiovasculares adversos em coronariopatas crônicos ao longo de dois anos.^12^

Estudar pacientes vulneráveis a doença aterosclerótica e até mesmo poder dispor de parâmetros de gravidade naqueles com doença já presente através de novos marcadores tem sido um desafio para as pesquisas médicas na atualidade. Nesse contexto, os novos biomarcadores conquistam um espaço importante, considerando-se o aspecto de morbidade e mortalidade da doença coronariana, podendo vir a ser preditores de eventos cardiovasculares e, assim, contribuir para prevenção, detecção precoce e rastreamento da DAC.

## Métodos

### Objetivo primário

Avaliar os níveis séricos dos biomarcadores interleucina 18 (IL-18) e proteína precursora do trombo (TpP) em pacientes com síndrome coronariana aguda ou crônica em relação ao grupo-controle.

### Objetivos secundários

Verificar a associação dos níveis séricos dos biomarcadores interleucina 18 (IL-18) e proteína precursora do trombo (TpP) com a cronicidade, agudização da doença arterial coronariana ou ausência de processo aterosclerótico coronariano obstrutivo.

Avaliar a relação dos dados epidemiológicos, antropométricos e fatores de risco da população estudada com os biomarcadores interleucina 18 (IL-18) e proteína precursora do trombo (TpP).

Nessa coorte transversal, foram estudados 119 pacientes, de ambos os sexos, maiores de 30 anos de idade, estratificados em três grupos: agudo (Grupo I – n=39), crônico (Grupo II – n=40) e controle (Grupo III – n=40), de acordo com os critérios de inclusão e exclusão estabelecidos para o estudo e comparados com o montante de dois biomarcadores: IL-18 e TpP.

Os pacientes pertencentes ao Grupo I (agudo) foram recrutados a partir de história clínica típica de síndrome coronariana aguda (IAMSST, IAMCSST e AI), acrescida dos dados eletrocardiográficos e enzimas cardíacas no momento da admissão. Uma vez identificados, foi coletado sangue em até 48 horas do início dos sintomas, para posterior dosagem dos biomarcadores. A seleção foi feita de forma sequencial, respeitando-se os critérios de inclusão, à medida que esses pacientes davam entrada na unidade hospitalar. Os pacientes pertencentes ao Grupo II foram selecionados de forma sequencial a partir da história clínica de DAC crônica comprovada por algum método de imagem (cateterismo cardíaco - CAT ou angiotomografia de corononária - angio-TCc) ou relato clínico de IAM prévio (com exames complementares comprobatórios – eletrocardiograma - ECG e marcadores miocárdicos). O Grupo III (controle) foi constituído por pacientes que apresentavam fatores de risco para DAC na ausência de lesão coronariana obstrutiva no momento da admissão ao banco de dados. A ausência de lesão coronariana aterosclerótica obstrutiva foi descartada através de cateterismo cardíaco ou angiotomografia de coronárias.

### Critérios de inclusão

Grupo I (agudo) – SCA (IAMCSST, IAMSSST e AI): a) IAM com ou sem supradesnivelamento de ST comprovado por curva de marcadores de necrose miocárdica positiva (troponina), associada ao ECG; b) AI a partir da clínica classicamente descrita e exames complementares (eletrocardiograma - ECG e cateterismo cardíaco - CAT); c) dosagem dos biomarcadores realizada nas primeiras 48 horas a partir do início dos sintomas.

Grupo II (crônico) – DAC crônica: a) comprovação por método de imagem (cateterismo cardíaco ou angiotomografia de coronárias) ou por história clínica de doença coronariana prévia – IAM prévio (diagnóstico com mais de seis meses comprovado por ECG e marcadores de necrose).

Grupo III (controle): a) presença ou não de um ou mais fatores de risco para DAC: HAS, dislipidemia (DLP), DM, sedentarismo, tabagismo, ex-tabagismo, insuficiência renal crônica, obesidade; b) exame de imagem cardíaca que comprove ausência de lesão coronariana obstrutiva – CAT ou angio-TCc – realizado nos últimos seis meses que antecederam a admissão ao banco de dados.

Foram adotados os seguintes critérios de exclusão:

- **Grupo I (agudo)** : a) revascularização miocárdica com menos de seis meses; b) insuficiência renal crônica em tratamento dialítico ou *clearence* <30 mL/min; c) doenças terminais; d) fração de ejeção prévia à SCA <40% (Simpson); e) não ter assinado o Termo de Consentimento Livre e Esclarecido (TCLE).
- **Grupo II (crônico)** : os mesmos do Grupo I, mais: a) DM descompensado; b) IAM com menos de seis meses; c) angina instável; d) angina estável de classe IV pela Sociedade Canadense Cardiovascular; d) classes funcionais III e IV pela New York Heart Association (NYHA).
- **Grupo III (controle)** : a) presença de qualquer lesão coronariana obstrutiva, mesmo que incipiente; b) insuficiência renal crônica em tratamento dialítico ou *clearence* <30 mL/min; c) doenças terminais; d) fração de ejeção <40% (pelo método de Simpson); e) angina estável; f) angina instável; g) IAM; h) DM descompensado; i) classes funcionais III e IV pela NYHA; j) não ter assinado o TCLE.

### Métodos de armazenamento e laboratoriais

Para dosagem da TpP foram coletados 4 mL de sangue venoso, colocado em tubo com citrato de sódio e centrifugado. Após centrifugação, foram retiradas duas alíquotas de 1 mL de plasma cada para serem congeladas a -80°C. A análise foi realizada posteriormente, através do método ELISA. Para dosagem da IL-18 foram coletados 8 mL de sangue venoso e colocado em tubo com sorogel. Após a coleta, aguardaram-se 30 min para retração do coágulo e posterior centrifugação. Após centrifugação, foram retiradas duas alíquotas de 1 mL de soro cada para serem congeladas a -80°C. A análise também foi realizada posteriormente pelo método ELISA.

### Análise estatística

Os dados obtidos foram analisados pelo programa estatístico SPSS (Statistical Package for the Social Sciences para Windows, versão 17.0, de 2008). Fixou-se em 0,05 ou 5% o nível de significância (p<0,05) e o intervalo de confiança em 95%. Foram utilizados os seguintes métodos estatísticos: análise de variância (ANOVA One-way F), utilizada para comparação das médias das variáveis que apresentaram distribuição normal e que tinham homogeneidade de variâncias pelo teste de Levene; teste de Tukey, usado como complementação da análise de variância, para comparação das médias das variáveis 2 a 2; teste do qui-quadrado (χ^2^ ), utilizado para comparação das distribuições de frequência das variáveis categóricas de amostras independentes. O *n* amostral desse estudo foi determinado por uma amostra de conveniência, porém seguiu a ordem de recrutamento, ou seja: os primeiros 39 pacientes em fase aguda das duas instituições participantes, os 40 primeiros crônicos e os 40 controles.

Este estudo foi aprovado pelos Comitês de Ética do HUPE e do INC, onde os pacientes foram recrutados, sob os números 2667/2010 CAAE: 0115.0.228.000-10 e 141.432/2012 CAAE: 09086412.9.1001.5272, respectivamente. Os participantes assinaram o TCLE antes de qualquer procedimento relacionado ao estudo.

## Resultados

A idade média foi de 59,5±9,7 anos (variação: 35 a 83 anos), sem diferença estatisticamente significativa entre os grupos. Nos Grupos I e II houve predomínio do sexo masculino e, no Grupo III, do feminino (p = 0,002). Em relação aos fatores de risco da amostra geral, chama atenção a alta incidência de sedentarismo, sobrepeso ou obesidade e hipertensão arterial em todos os grupos.

A Tabela 1 apresenta os fatores de risco para DAC entre os grupos estudados. Observou-se que apenas dislipidemia, ex-tabagismo e sedentarismo apresentaram diferença estatisticamente significativa. Comparado aos demais grupos, o Grupo II apresentou maior prevalência de indivíduos dislipidêmicos; o Grupo III apresentou maior prevalência de indivíduos não tabagistas que os outros grupos; e o Grupo I apresentou maior prevalência de indivíduos sedentários, em comparação aos demais. Não houve relação entre os fatores de risco, etnia ou sexo com os resultados obtidos com os marcadores em todos os grupos analisados.

**Tabela 1:** – Fatores de risco para DAC entre os grupos estudados

Fatores de risco								

	Grupo I		Grupo II		Grupo III		Teste estatistico	p

	n	%	n	%	n	%		
**HAS**								
Sim	31	79,5	28	70,0	32	80,0	χ^2^=1,4	0,495
Não	8	20,5	12	30,0	8	20,0		
**DLP**								
Sim	16	41,0	32	80,0	18	45,5	χ^2^=14,8	0,001
Não	23	59,0	8	20,0	22	55,0		
**DM**								
Sim	12	30,8	10	25,0	8	20,0	χ^2^=1,2	0,544
Não	27	69,2	30	75,0	32	80,0		
**TAB**								
Sim	3	7,7	5	12,5	0	0,0	χ^2^=5	0,079
Não	36	92,3	35	87,5	40	100,0		
**Ex-TAB**								
Sim	15	38,5	22	55,0	7	17,5	χ^2^=12,1	0,002
Não	24	61,5	18	45,0	33	82,5		
**SEDENT**								
Sim	38	97,4	31	77,5	31	77,5	χ^2^=7,7	0,021
Não	1	2,6	9	22,5	13	22,5		
**SOB/OBES**								
Sim	30	76,9	31	77,5	27	67,5	χ^2^=1,3	0,521
Não	9	23,1	9	22,5	13	32,5		
**IRC**								
Sim	2	5,1	2	5,0	0	0,0	χ^2^=2,0	0,351
Não	37	94,9	38	95,0	40	100,0		

Grupo I: pacientes agudos (SCA); Grupo II: pacientes crônicos (DAC); Grupo III: grupo-controle (com Risk factors para DAC, mas sem lesão coronariana); DAC: doença arterial coronariana; HAS: hipertensão arterial sistêmica; DLP: dislipidemia; DM: diabetes melito; TAB: tabagismo; Ex-TAB: ex-tabagismo; SEDENT: sedentarismo; SOB/OBES: sobrepeso/obesidade; IRC: insuficiência renal crônica. Corrigido pelo teste exato de Fisher.

Os valores médios dos marcadores IL-18 e TpP intergrupos e intragrupos são mostrados na Tabela 2 .

**Tabela 2:** – Valores médios dos biomarcadores IL-18 e TpP dos grupos estudados

Biomarcadores	Grupo I	Grupo II	Grupo III	Teste estatístico	p	Comp 2 a 2
IL-18 (pg/ml)	1325,44	353,81	633,25	F=6.61	0,002	GI>GII
	(±1860,13)	(±273,65)	(±993,93)			GII=GIII
						GI>GIII
TpT (µg/mL)	35,86±28,36	25,66±12,17	18,0±8,45	F=9,31	0,000	GI>GII
						GII=GIII
						GI>GIII

Em relação às diferenças dos valores médios de IL-18 entre os grupos, observou-se que o Grupo I apresentou valores maiores que os dos demais grupos (1325,44±1860,13 pg/mL), com significância estatística. Na comparação dois a dois, o Grupo I apresentou média maior que os Grupos II (353,81±273,65 pg/mL) e III (633,25±993,93 pg/mL), porém os Grupos II e III apresentaram médias estatisticamente iguais. Os pacientes com instabilidade da doença aterosclerótica apresentaram níveis maiores de IL-18 quando comparados àqueles que tinham lesão coronariana estável ou àqueles que tinham fatores de risco para DAC na ausência de processo aterosclerótico coronariano, o que nos possibilita inferir que o aumento das concentrações de IL-18 pode estar associado a instabilidade da placa aterosclerótica.

Em relação ao biomarcador TpP entre os grupos, observaram-se valores maiores no Grupo I (35,86±28,36 µg/mL, respectivamente, p<0,001) quando comparados aos demais grupos. Na comparação dois a dois, o Grupo I também apresentou média maior que a dos Grupos II (25,66±12,17 µg/mL) e III (18,0±8,45 µg/mL); porém, quando se compararam os Grupos II e III, observou-se que as médias foram estatisticamente iguais ( Tabela 2 ).

## Discussão

A identificação de indivíduos assintomáticos portadores de aterosclerose é fundamental para se instituírem medidas de tratamento e prevenção secundária, como também aqueles com possível evolução mais desfavorável.

Mallat et al.^13^ evidenciaram que as concentrações de IL-18 no plasma estão aumentadas nos pacientes com SCA com ou sem necrose miocárdica, ressaltando também que as concentrações se correlacionam à gravidade da disfunção miocárdica. Estudaram uma casuística de 53 pacientes, admitidos em unidade cardiointensiva com dor torácica e alteração do segmento ST, de forma sequencial; após análise da troponina, esses pacientes foram selecionados para o grupo de AI ou de IAM. Os pacientes pertencentes ao grupo de AI apresentaram valor médio de IL-18 de 214,7 (116,6 a 297,0) pg/mL, e os pertencentes ao grupo de IAM apresentaram valor médio de 164,6 (53,6 a 602,5) pg/mL. Esses pacientes foram comparados a mais dois grupos: um com doença arterial coronariana estável ( *n* =9) e outro grupo ( *n* =11) sem lesão coronariana (grupo-controle). Observou-se que os valores médios das concentrações de IL-18 no grupo-controle (46,8 [34,2 a 68,2] pg/mL) foram significativamente diferentes daqueles dos pacientes com angina estável (85,7 [56,0 a 157,7] pg/mL, p<0,01), sugerindo que as concentrações de IL-18 em pacientes com doença arterial coronariana estável podem estar associadas à presença de doença arterial coronariana avançada. No grupo de pacientes com angina instável, as concentrações médias de IL-18 foram significativamente maiores do que no grupo-controle (p<0,001) ou no grupo com angina estável (p=0,001). No grupo de pacientes com IAM, as concentrações médias de IL-18 também foram significativamente superiores às do grupo-controle (p<0,001) ou do grupo com angina estável (p<0,01). As concentrações de IL-18 não diferiram significativamente entre o grupo com angina instável e o grupo com IAM.^13^ No mesmo estudo, observou-se que as concentrações séricas de IL-18 estão significativamente correlacionadas à gravidade da disfunção miocárdica, avaliadas pela determinação da fração de ejeção (FE) ventricular; a FE média entre os grupos foi de 55% e os valores médios da IL-18 foram de 46,8 pg/mL para pacientes sem lesão coronariana, 85,7 pg/mL para pacientes com angina estável, 214 pg/mL para aqueles com angina instável e 164,6 pg/mL para pacientes com IAM. Na comparação com os resultados encontrados neste estudo, observou-se que a FE média entre os grupos foi de 65,09%, e os valores médios da concentração de IL-18 foram maiores no Grupo I quando comparados aos do Grupo II e aos do Grupo III, porém nesta população ( *n* =119) obteve-se distribuição da função sistólica dentro da normalidade para todos os grupos, sendo que o Grupo I apresentou discreta redução da prevalência de indivíduos com FE normal, sem significado estatístico. Também se observou que as concentrações de IL-18 entre os pacientes com DAC crônica e sem lesão coronariana foram estatisticamente equivalentes, não se replicando os resultados anteriores.

Blankenberg et al.^14^ evidenciaram em seu estudo prospectivo, realizado com uma coorte de 1229 pacientes com doença coronariana documentada e acompanhados pelo tempo médio de 3,9 anos, que 95 pacientes morreram por causas cardiovasculares e que as concentrações médias de IL-18 no soro foram significativamente maiores nos pacientes que tiveram evento cardiovascular fatal do que naqueles que não apresentaram tal evento (68,4 pg/mL × 58,7 pg/mL, p<0,001). Nesse estudo foram incluídos pacientes com angina estável ( *n* =855) e pacientes com angina instável ( *n* =373) e avaliado o valor preditivo da IL-18 em relação a morte cardiovascular. Observou-se que, em ambos os grupos, houve nítido aumento do risco de eventos cardiovasculares fatais de acordo com o valor médio da IL-18, porém o grupo dos pacientes com angina instável apresentou valor mais elevado desse marcador. Os autores chamam atenção para essa última análise, pois o grupo de angina instável tem um tamanho amostral menor do que o grupo dos pacientes com angina estável; portanto, o nível de IL-18 no soro pode ser identificado como forte preditor independente de morte por causas cardiovasculares em pacientes com DAC, independentemente do estado clínico à admissão. Esse resultado corrobora fortemente as evidências experimentais de inflamação mediada pela IL-18, possibilitando a aceleração e vulnerabilidade da aterosclerose.^14^

Estudos evidenciam que o aumento da IL-18 no soro está associado também a alguns fatores de risco, como diabetes melito (DM) tipo 2 e síndrome metabólica, além da gravidade da aterosclerose.^15^

Suchanek et al.^16^ analisaram o aumento das concentrações de IL-18 no soro em pacientes com DAC e DM tipo 2. Os autores avaliaram um grupo de 130 pacientes com DAC avançada (pelo menos duas lesões coronarianas, uma delas com estenose >70%), tendo sido selecionados 43 com diagnóstico prévio de DM em tratamento e outro grupo com 31 pacientes saudáveis (grupo-controle). Os grupos foram pareados de forma semelhante para idade, IMC, DLP, tabagismo; observou-se maior concentração de IL-18 em pacientes com DAC (463,48±111,7 pg/mL) quando comparados ao grupo-controle (248,99±103,69 pg/mL), porém os pacientes com DAC e DM apresentaram maior concentração de IL-18 quando comparados aos pacientes com DAC sem DM (500 pg/mL × 430 pg/mL, p=0,04). Os mecanismos responsáveis pela elevação da IL-18 em pacientes diabéticos não foram totalmente esclarecidos, mas acredita-se que controle glicêmico deficiente, nefropatia diabética, obesidade e inflamação sejam considerados possíveis causas para justificar o aumento das concentrações séricas de IL-18 nesse perfil de pacientes.^16^ No presente estudo, não se observou diferença estatística significativa quando se comparou o fator de risco DM entre os grupos de pacientes associado ao nível sérico de IL-18, evidenciando que as amostras de pacientes com SCA, DAC crônica e pacientes saudáveis eram semelhantes entre si.

Em estudo observacional semelhante a este, porém com análise prospectiva, 194 pacientes, sendo 75 agudos e 119 crônicos, foram comparados a 68 controles. A análise de células da musculatura lisa da aorta desses pacientes revelou valores maiores para os coronariopatas do que para os controles, e que a IL-18 pode ser um fator de risco independente para DAC.^17^

Em outro estudo com 118 pacientes coronariopatas submetidos a estudo angiográfico, composto por 67 em fase aguda e 51 com angina *pectoris* estável, os valores da IL-18 foram significativamente maiores entre os agudos em comparação aos crônicos, o que está em acordo com os resultados aqui apresentados.^18^

Em relação à formação do trombo, Goetze evidencia a importância do aumento das concentrações de TpP nos pacientes com DAC, destacando a relevância de se ter um marcador de trombose em atividade para fornecimento de informações importante na SCA em curso.^19^

Nesta mesma linha, Mega et al.^20^ destacaram o valor prognóstico da TpP em pacientes com SCA, evidenciando que níveis aumentados de TpP estão associados a maior risco de morte e complicações isquêmicas, deixando claro que a incorporação de um marcador de coagulação ativada, como a TpP, em pacientes com doença cardiovascular estabelecida e com fatores de risco para DAC pode oferecer uma análise complementar valiosa na avaliação de risco de SCA. Realizado com 284 pacientes saudáveis e 2349 pacientes com SCA, esse estudo observou que a concentração média de TpP foi maior nos pacientes com SCA (8,9 µg/mL × 3,6 µg/mL, p<0,001), estando este fato correlacionado a pior prognóstico.^20^ No presente estudo, observou-se que a dosagem de TpP foi significativamente maior nos pacientes com diagnóstico de SCA do que naqueles com DAC crônica e no grupo-controle (p<0,001). No entanto, por se tratar de estudo observacional, não houve avaliação prognóstica desses pacientes, o que impossibilitou melhor análise dos desfechos clínicos que pudessem estar correlacionados; faz parte do planejamento, contudo, a avaliação desses dados.

Laurino et al.^21^ estudaram uma coorte de 115 pacientes com sintomas sugestivos de IAM com menos de 6 horas de duração a partir do início da dor. Foram dosadas amostras de sangue dos pacientes em 0, 1, 2, 4, 8, 16 e 24 horas após a apresentação para alguns biomarcadores: creatina quinase total (CK total), CK-MB, mioglobina, troponina I e TpP. Os autores observaram aumento significativo das concentrações séricas de TpP em 15 dos 17 pacientes com IAM diagnosticado em 6 horas após início dos sintomas (p<0,001); em 2 dos 8 pacientes que apresentaram IAM após 6 horas do início dos sintomas (p<0,008); em 22 dos 35 pacientes com angina instável (p<0,001); e em 15 dos 30 pacientes com angina estável (p<0,001) e 3 dos 5 pacientes com fibrilação atrial (p<0,001); em 6 dos 9 pacientes com insuficiência cardíaca congestiva (p<0,001); e em 6 dos 11 pacientes com dor torácica não cardíaca (p<0,001). As concentrações máximas de TpP em pacientes com IAM precederam as dos outros marcadores em 2 a 4 horas.^21^ Portanto, a trombose aguda está associada a alterações clínicas significativas, incluindo o IAM. Um teste preciso, rápido e confiável para detecção do trombo seria uma ferramenta inestimável para os médicos no diagnóstico, monitoramento e tratamento dos pacientes com dor torácica aguda.^22^

Estudos evidenciam o valor da TpP na mensuração do processo agudo trombótico, destacando a SCA, e outros relacionam os valores aumentados desse biomarcador na presença de alguns fatores de risco para DAC, tais como HAS e DM, em pacientes sem evidências de SCA. Por favorecerem um estado de hipercoagulabilidade, esses fatores de risco podem estar relacionados a um efeito pró-trombótico e desencadear um processo trombogênico ativo. A presença de DM tipo 2 pode estar associada a aumento dos valores séricos de TpP devido à predisposição desse perfil de pacientes para apresentar disfunção endotelial marcadamente aumentada, além de maior ativação sistêmica da coagulação, provocando alterações transitórias e favorecendo eventos cardíacos agudos.^22 , 23 , 24^ A presença de HAS também pode conferir um meio hipercoagulável e pró-trombótico e favorecer descompensação aguda da doença arterial coronariana.^23 , 24^ No presente estudo, observou-se que o DM e a HAS não conferiram diferença estatisticamente significativa entre os grupos estudados, e não tiveram influência no resultado final da dosagem de TpP em relação às diferentes fases da doença coronariana (presença de fator de risco, DAC crônica e SCA). Portanto, o resultado obtido não sofreu influência desses fatores de risco.

### Limitações do estudo

O *n* amostral é pequeno, representando uma amostra de conveniência, que pode ser atribuído a dois fatores: a seleção dos pacientes realizada em hospitais que não apresentavam unidade de emergência aberta, dificultando a formação do grupo de pacientes agudos. Na análise das médias encontradas de IL-18 e TpP, verificou-se igualdade entre o grupo de pacientes crônicos e o grupo-controle. Fazendo uma avaliação crítica dessa observação, pode-se destacar que não houve realização de exames complementares (p. ex., Doppler de carótidas e vertebrais) ou ultrassonografia intracoronariana para descartar processo aterosclerótico em outros locais, o que poderá ter influenciado nesse resultado, não possibilitando melhor avaliação dos biomarcadores com a cronicidade ou ausência de aterosclerose. Fração de ejeção normal em todos os grupos excluiu pacientes mais graves, o que pode ter influenciado nos resultados. No presente estudo, apesar de se observar que os valores médios de IL-18 e de TpT no soro estavam mais elevados no grupo de pacientes agudos, quando comparado ao grupo com angina estável ou sem lesão coronariana, não se pode fazer uma avaliação prognóstica, tem como objetivo estimular continuidade da pesquisa para confirmar a hipótese e pretendendo fazer acompanhamento anual dos sujeitos desta amostra.

## Conclusões

As concentrações médias de IL-18 e TpP encontram-se elevadas na fase aguda da doença arterial coronariana, sugerindo correlação desse biomarcador com a agudização e instabilidade da síndrome coronariana aguda.

As concentrações médias de IL-8 e TpP foram menores na fase crônica da doença coronariana e nos pacientes do grupo-controle; entretanto, neste estudo observou-se que o grupo crônico e o grupo-controle apresentaram médias estatisticamente equivalentes.

Os resultados não sofreram influência das diferenças de sexo e etnia dos grupos.
