# Supplementary material for: Papel da Interleucina 18 e da Proteína Precursora do Trombo na Doença Arterial Coronariana
Source: Arq Bras Cardiol. 2020 May 12;114(4):692–8. [Article in Portuguese] doi: 10.36660/abc.20190176 (PMC9744352; doi:10.36660/abc.20190176)
Supplement: Translation

# Translation: Role of Interleukin-18 and the Thrombus Precursor Protein in Coronary Artery Disease

**Authors:** Carlos Scherr, Denilson Campos de Albuquerque, Roberto Pozzan, Kezia Ataide, Talita Ludmila, Fernanda Blanco, Claudio Martins Mangia

**Affiliations:** 1 Brazil Ministério da Saúde – Cardiologia, Brasília, DF - Brazil; 2 Brazil Universidade do Estado do Rio de Janeiro – Cardiologia, Rio de Janeiro, RJ - Brazil; 3 Brazil Universidade do Estado do Rio de Janeiro – Cardiologia/Hipertensão, Rio de Janeiro, RJ - Brazil; 4 Brazil Hospital Universitário Pedro Ernesto – Cardiologia, Rio de Janeiro, RJ - Brazil; 5 Brazil Fundação Pró Coração – Cardiologia, Rio de Janeiro, RJ – Brazil

**Keywords:** Cardiovascular Diseases/mortality, Coronary Artery Diseases, Interleukin 18, Biomarkers, Acute Coronary Syndrome/prevention and control

## Abstract

**Background:**

Coronary failure is the leading cause of death worldwide and identifying patients at higher risk for coronary artery disease (CAD) is a challenge.

**Objectives:**

To test the biomarkers interleukin 18 (IL-18) and thrombus precursor protein (TpP), involved in atherogenesis, to aid in the early assessment of CAD.

**Methods:**

This was a cross-sectional cohort of 119 patients, stratified into three groups: Group I - acute coronary syndrome (39); Group II - chronic CAD (40) and Group III - control, without coronary lesion, but who might have risk factors for CAD (40). Statistical analysis was performed using the statistical program SPSS (Statistical Package for the Social Sciences) for Windows ,version 17.0 of 2008. The significance level was set at 0.05 or 5% (p <0.05), with a 95% confidence interval. Chi-square test (χ2), Analysis of variance (ANOVA), and Tukey’s test were used.

**Results:**

The mean age was 60.36 ± 9.64 years; there was a prevalence of females in Group III (65.0% p = 0.002), but without statistical significance for the means of IL-18 and TpP. The means of IL-18 and TpP were increased in Group I when compared to the other groups; IL-18 = 1325.44 ± 1860.13 ng/dL, p = 0.002; TpP = 35.86 ± 28.36 µg / mL, p <0.001). When compared two-by-two, it was observed that Group I had higher mean IL-18 and TpP values than Group II (IL-18 = 353.81 ± 273.65 ng / dL; TpP = 25.66 ± 12, 17 µg / mL) and Group III (IL-18 = 633.25 ± 993.93 ng / dL; TpP = 18.00 ± 8.45 µg / mL).

**Conclusion:**

There was an increase in these biomarkers in acute CAD, suggesting a relationship with the atherosclerotic plaque instability process, but not with the chronic phase. (Arq Bras Cardiol. 2020; 114(4):692-698)

## Introduction

Coronary artery disease is one of the main manifestations of cardiovascular disease in the 21^st^century, and its importance lies in its high morbidity and mortality. The identification of patients at higher risk becomes necessary to contribute to the improvement of this condition and rationalize costs.^1^ Modern medicine has developed rapidly in the field of prevention, early detection and screening of diseases, not being restricted to treatment.

Therefore, early detection associated with the immediate treatment of cardiovascular disease (CVD) has become one of the most challenging tasks for doctors and researchers worldwide. Several biomarkers have been studied in recent years in the diagnosis, prognosis, prediction of adverse events and therapeutic monitoring.^2^ However, the first initiative towards prevention is to apply strategies to identify the individual likely to have atherosclerotic events. In this sense, in addition to the widely known risk factors contributing to the process of identifying vulnerable individuals, the use of biomarkers involved in atherogenesis can be a key factor in the risk assessment of coronary artery disease (CAD).^3 , 4^

Atherosclerosis is a chronic, multifactorial, slow and progressive inflammatory disease, resulting from several specific cell and molecular responses that lead to endothelial aggression, affecting mainly the intima layer of medium and large-caliber arteries.

Generally, the rupture of the fibrous capsule of the atherosclerotic plaque leads to thrombosis, which to a lesser extent can result from superficial endothelial erosion. Ruptured plaques are usually associated with inflammation of the intima and adventitia, intra-plaque hemorrhage, exposure of thrombogenic material to the bloodstream, triggering platelet accumulation, coagulation cascade activation and fibrin deposition.^5^

The inflammatory response in atherogenesis consists of functional alterations in endothelial cells, T-lymphocytes, monocyte-derived macrophages, smooth muscle cells and, in the early stages, it is caused by the accumulation of lipids in the arterial walls.^6^ The activation of these cells triggers the formation and interaction of several cytokines, adhesion molecules, growth factors, accumulation of lipids and proliferation of smooth muscle cells. In addition to these factors, the inflammatory response can be induced by oxidative stress (oxidation of low-density lipoprotein - LDL).^7 , 8^

Currently, new biomarkers are being studied in association with acute or chronic coronary syndrome and correlated with the prognostic value, which is the objective of this study.

Two of them were evaluated in this study: the thrombus precursor protein (TpP) and interleukin 18 (IL-18) in individuals with acute myocardial infarction with and without ST-elevation, unstable angina (UA), chronic coronary artery disease and individuals who had no evidence of obstructive coronary atherosclerotic disease, but in the presence of risk factors for CAD. The choice of biomarkers was based on their involvement with the inflammatory (IL-18) and thrombotic (TpP) processes, within the context of atherosclerosis and aggravation of atherosclerotic disease.

TpP is a biomarker used to estimate soluble fibrin polymers. Elevated levels of TpP indicate a pro-thrombotic status and active thrombogenesis. In patients with ACS, increased levels of this biomarker are associated with a higher risk of death and ischemic complications; therefore, TpP has been shown to be an independent marker for adverse cardiovascular outcomes.^9^ The IL-18 biomarker is a pro-inflammatory cytokine, closely associated with atherosclerotic plaque instability, and is therefore a good predictor of undesirable events in ACS.^10 , 11^ In an observational study, it was also shown to be a good predictor of adverse cardiovascular events in chronic coronary disease over two years.^12^

Studying patients vulnerable to atherosclerotic disease and even being able to have severity parameters in those with the disease already present through new markers has been a challenge for current medical research. In this context, the new biomarkers occupy an important space, considering the morbidity and mortality aspect of coronary disease, which may become predictors of cardiovascular events and thus contribute to the prevention, early detection and screening of CAD.

## Methods

### Primary endpoint

To evaluate the serum levels of biomarkers: interleukin 18 (IL-18) and thrombus precursor protein (TpP) in patients with acute or chronic coronary syndrome in relation to the control group.

### Secondary endpoints

To verify the association of serum levels of biomarkers interleukin 18 (IL-18) and thrombus precursor protein (TpP) with disease chronicity, worsening of coronary artery disease or absence of obstructive coronary atherosclerotic process. To evaluate the association between epidemiological, anthropometric data and risk factors of the studied population with the biomarkers interleukin 18 (IL-18) and thrombus precursor protein (TpP).

In this cross-sectional cohort, 119 patients, of both genders, older than 30 years of age, were stratified into three groups: acute (Group I - n = 39), chronic (Group II - n = 40) and control (Group III - n = 40), according to the inclusion and exclusion criteria established for the study and compared with the quantitative analysis of two biomarkers: IL-18 and TpP.

Patients belonging to Group I (acute) were recruited based on the typical clinical history of acute coronary syndrome (NSTEMI, STEMI, and UA), plus electrocardiographic data and cardiac enzymes at admission. Once identified, blood was collected within 48 hours of symptom onset, for subsequent biomarker measurement. The selection was made sequentially, according to the inclusion criteria, as these patients were admitted to the hospital. Patients belonging to Group II were selected sequentially based on the clinical history of chronic CAD confirmed by some imaging method (cardiac catheterization - CAT or coronary angiotomography - Angio-CT) or clinical report of previous AMI (with complementary exams demonstrating the condition - electrocardiogram - ECG and myocardial markers). Group III (control) consisted of patients who had risk factors for CAD in the absence of obstructive coronary lesion at the time of admission to the database. The absence of atherosclerotic obstructive coronary lesion was ruled out through cardiac catheterization or coronary angiotomography.

Inclusion criteria

- **Group I (acute) -** ACS (NSTEMI, STEMI and UA) a) AMI with or without ST-elevation demonstrated by the positive curve of myocardial necrosis markers (troponin), associated with ECG. b) UA based on the classically described clinical picture and complementary exams (electrocardiogram - ECG and cardiac catheterization - CAT). c) Measurement of biomarkers performed within the first 48 hours of symptom onset.
- **Group II (chronic) -** chronic CAD a) confirmation by imaging method (cardiac catheterization or coronary angiotomography) or with a clinical history of previous coronary disease - previous AMI (diagnosis over six months before, confirmed by ECG and necrosis markers).
- **Group III (control)** a) Presence or not of one or more risk factors for CAD: SAH, dyslipidemia (DLP), diabetes mellitus (DM), physical inactivity, smoking, ex-smoker, chronic renal failure, obesity. b) Cardiac imaging examination that proved the absence of obstructive coronary lesion: CAT or angio-CT, performed in the last six months prior to admission to the database.

The following exclusion criteria were adopted:

**Group I (acute):** a) coronary artery bypass grafting surgery performed less than six months before. b) Chronic renal failure undergoing dialysis or clearance <30 mL / min. c) Terminal illnesses. d) Ejection fraction prior to ACS <40% (Simpson); e) Not signing the Free and Informed Consent (FIC) form.

**Group II (chronic):** the same as group I plus, a) decompensated DM. b) AMI that occurred less than six months before. c) Unstable angina. d) Class IV stable angina according to the Canadian Cardiovascular Society. d) Functional class III and IV according to the New York Heart Association (NYHA).

**Group III (control)** : a) presence of any obstructive coronary lesion, even if incipient. b) Chronic renal failure undergoing dialysis or clearance <30 mL / min. c) Terminal illnesses. d) Ejection fraction <40% (using the Simpson method). e) Stable angina. f) Unstable angina. g) AMI. h) Decompensated DM. i) NYHA functional class III and IV. j) Not signing the FIC form.

### Storage and laboratory methods

For the measurement of TpP, 4 mL of venous blood were collected, placed in a tube with sodium citrate and centrifuged. After centrifugation, two 1-mL aliquots of plasma were removed to be frozen at -80 °C. The analysis was carried out later using the ELISA method. For the measurement of IL-18, 8 mL of venous blood were collected and placed in a serum gel tube. After collection, we waited 30 min for clot retraction and subsequent centrifugation. After centrifugation, two 1-mL aliquots of serum were removed to be frozen at -80 °C. The analysis was also performed later using the ELISA method.

### Statistical analysis

The data obtained were analyzed using the statistical program SPSS (Statistical Package for the Social Sciences for Windows), version 17.0 of 2008. The level of significance was set at 0.05 or 5% (p <0.05) and the confidence interval at 95%. The following statistical methods were used: Analysis of variance (One-way ANOVA F): used to compare the means of variables that showed normal distribution and had homogeneity of variances by the Levene test. Tukey’s test: used as a complement to the analysis of variance, to compare the means of variables 2 by 2. Chi-square test ( **χ** ^2^): used to compare the frequency distributions of the categorical variables from independent samples. This study sample size was determined by a convenience sample; however, it followed the order of recruitment, that is, the first 39 patients in the acute phase of the two participating institutions, the first 40 chronic patients and the 40 controls.

This study was approved by the Ethical Committees of HUPE and INC, where patients were recruited, under numbers 2667/2010 CAAE: 0115.0.228.000-10 and 141.432/2012 CAAE: 09086412.9.1001.5272, respectively. The participants signed the Free and Informed Consent (FIC) form before any procedure related to the study was performed.

## Results

The mean age was 59.5 ± 9.7 years (range: 35-83 years), with no statistically significant difference between the groups. In groups I and II there was a predominance of males and in Group III, of females (p = 0.002).

Regarding the risk factors of the overall sample, it is noteworthy the high incidence of sedentary lifestyle, overweight / obesity and arterial hypertension in all groups.

Table 1 shows the risk factors for CAD between the studied groups. It was observed that only dyslipidemia, being an ex-smoker and physical inactivity showed a statistically significant difference. Group II had a higher prevalence of dyslipidemic individuals when compared to the other groups; Group III had a higher prevalence of non-smoking individuals than the other groups; and Group I had a higher prevalence of sedentary individuals compared to the others. There was no association between risk factors, ethnicity or gender with the results obtained of the markers in all analyzed groups.

**Table 1:** – Risk factors for CAD in the study groups

Risk factors								

	Group I		Group II		Group III		Statistic test	p

	n	%	n	%	n	%		
**SAH**								
Yes	31	79.5	28	70.0	32	80.0	χ^2^=1.4	0.495
No	8	20.5	12	30.0	8	20.0		
**DLP**								
Yes	16	41.0	32	80.0	18	45.5	χ^2^=14.8	0.001
No	23	59.0	8	20.0	22	55.0		
**DM**								
Yes	12	30.8	10	25.0	8	20.0	χ^2^=1.2	0.544
No	27	69.2	30	75.0	32	80.0		
**SMOKER**								
Yes	3	7.7	5	12.5	0	0.0	χ^2^=5	0.079
No	36	92.3	35	87.5	40	100.0		
**EX-SMOKER**								
Yes	15	38.5	22	55.0	7	17.5	χ^2^=12.1	0.002
No	24	61.5	18	45.0	33	82.5		
**SEDENT**								
Yes	38	97.4	31	77.5	31	77.5	χ^2^=7.7	0.021
No	1	2.6	9	22.5	13	22.5		
**OVER/OBES**								
Yes	30	76.9	31	77.5	27	67.5	χ^2^=1.3	0.521
No	9	23.1	9	22.5	13	32.5		
**CRF**								
Yes	2	5.1	2	5.0	0	0.0	χ^2^=2.0	0.351
No	37	94.9	38	95.0	40	100.0		

*Grupo I: pacientes agudos (SCA); Grupo II: pacientes crônicos (DAC); Grupo III: grupo-controle (com Risk factors para DAC, mas sem lesão coronariana); DAC: doença arterial coronariana; HAS: hipertensão arterial sistêmica; DLP: dislipidemia; DM: diabetes melito; TAB: tabagismo; Ex-TAB: ex-tabagismo; SEDENT: sedentarismo; SOB/OBES: sobrepeso/obesidade; IRC: insuficiência renal crônica. Corrigido pelo teste exato de Fisher.*

Mean of intergroup and intragroup IL-18 and TpP markers in table 2

**Table 2:** – Means of the IL-18 and TpP biomarkers of the study groups

Biomarkers	Group I	Group II	Group III	Statistic test	p	Comp. 2 by 2
IL-18 (pg/ml)	1325.44	353.81	633.25	F=6.61	0.002	GI>GII
	(±1860.13)	(±273.65)	(±993.93)			GII=GIII
						GI>GIII
TpT (µg/mL)	35.86±28.36	25.66±12.17	18.0±8.45	F=9.31	0.000	GI>GII
						GII=GIII
						GI>GIII

Regarding the differences in the means of IL-18 levels between the groups, it was observed that Group I had higher values than the other groups (1325.44 ± 1860.13 pg / mL), with statistical significance. In the two-by-two comparison, Group I had a higher mean than Groups II (353.81 ± 273.65 pg / mL) and III (633.25 ± 993.93 pg / mL), but Groups II and III showed statistically equal means. Those with instability of atherosclerotic disease had higher levels of IL-18 when compared to patients who had stable coronary lesion or those who had risk factors for CAD in the absence of a coronary atherosclerotic process, allowing us to infer that the increase in IL-18 levels is associated with atherosclerotic plaque instability.

Regarding the TpP biomarker between the groups, higher values were observed in Group I (35.86 ± 28.36 µg / mL), respectively, p <0.001) when compared with the other groups. In the two-by-two comparison, Group I also had a higher mean than Groups II (25.66 ± 12.17 µg / mL) and III (18.0 ± 8.45 µg / mL); however, when Groups II and III were compared, it was observed that the means were statistically equal ( Table 2 ).

## Discussion

The identification of asymptomatic individuals with atherosclerosis is essential to implement secondary treatment and prevention measures, as well as those with a possibly more unfavorable evolution.

Mallat et al.^13^ showed that plasma concentrations of IL-18 are increased in patients with ACS with or without myocardial necrosis, also pointing out that the concentrations correlate with the myocardial dysfunction severity. They studied a sample of 53 patients, admitted to a cardio-intensive unit with chest pain and alteration of the ST-segment in a sequential manner; after troponin analysis, they were selected for the UA or AMI group. The patients belonging to the UA group had a mean IL-18 level of 214.7 (116.6-297.0) pg / mL, and those belonging to the AMI group had a mean of 164.6 (53.6-602.5) pg / mL. These patients were compared to two more groups: one with stable coronary artery disease (n = 9) and another group (n = 11) without coronary lesions (control group). It was observed that the mean IL-18 levels in the control group (46.8 (34.2-68.2) pg / mL) were significantly different from those with stable angina (85.7 (56.0- 157.7) pg / mL, p <0.01), suggesting that IL-18 concentrations in patients with stable coronary artery disease may be associated with the presence of advanced coronary artery disease. In the group of patients with unstable angina, the mean IL-18 levels were significantly higher than in the control group (p <0.001) or in the group with stable angina (p = 0.001). In the group of patients with AMI, the mean IL-18 levels were also significantly higher than in the control group (p <0.001) or the group with stable angina (p <0.01). IL-18 levels did not significantly differ between the group with unstable angina and the group with AMI.^13^ In this same study, it was observed that serum IL-18 levels were significantly correlated with myocardial dysfunction severity, assessed by determining the ventricular ejection fraction (EF); the mean EF between the groups was 55% and mean IL-18 values were 46.8 pg / mL for non-coronary patients, 85.7 pg / mL for patients with stable angina, 214 pg / mL for those with unstable angina and 164.6 pg / mL for patients with acute myocardial infarction. Comparing with the results found in this study, it was observed that the mean EF between the groups was 65.09% and the mean levels of IL-18 were higher in Group I when compared to Group II and Group III; however, in this population (n = 119), systolic function distribution within normality was obtained for all groups, and Group I showed a slight reduction in the prevalence of individuals with normal EF, without statistical significance. It was also observed that the levels of IL-18 in patients with chronic CAD and without coronary lesions were statistically equivalent, not replicating the previous results.

Blankenberg et al.^14^ showed in their prospective study carried out with a cohort of 1229 patients with documented coronary heart disease and followed for an average of 3.9 years, that 95 patients died from cardiovascular causes and the mean IL-18 serum concentrations were significantly higher in patients with a fatal cardiovascular event than the ones without it (68.4 pg / mL vs. 58.7 pg / mL, p <0.001). This study included patients with stable angina (n = 855) and patients with unstable angina (n = 373) and the predictive value of IL-18 in relation to cardiovascular death was evaluated. It was observed that both groups showed an evident increase in the risk of fatal cardiovascular events according to the mean value of IL-18; however, the group of patients with unstable angina showed a higher value of this marker. The authors draw attention to this last analysis, since the group with unstable angina had a smaller sample size than the group of patients with stable angina; thus, the serum level of IL-18 can be identified as a strong independent predictor of death from cardiovascular causes in patients with CAD, regardless of the clinical status at admission. This result strongly supports the experimental evidence of IL-18-mediated inflammation, allowing the acceleration and vulnerability of atherosclerosis.^14^

Studies have shown that the increase in serum IL-18 levels is also associated with some risk factors, such as type 2 DM and metabolic syndrome, in addition to the atherosclerosis severity.^15^

Suchanek et al.^16^ analyzed the increase in serum IL-18 levels in patients with CAD and type 2 DM. The authors evaluated a group of 130 patients with advanced CAD (at least two coronary lesions, one with stenosis >70%), and 43 were selected, with a previous diagnosis of DM undergoing treatment and another group with 31 healthy patients (control group). The groups were similarly matched for age, BMI, DLP, smoking; a higher level of IL-18 was observed in patients with CAD (463.48 ± 111.7 pg / mL) when compared to the control group (248.99 ± 103.69 pg / mL), but patients with CAD and DM showed a higher level of IL-18 when compared to CAD patients without DM (500 pg / mL vs. 430 pg / mL, p = 0.04). The mechanisms responsible for the increase in IL-18 levels in diabetic patients are yet to be fully clarified; however, it is believed that deficient glycemic control, diabetic nephropathy, obesity and inflammation are considered possible causes to justify the increase in serum IL-18 levels in this patient profile.^16^ In the present study, there was no statistically significant difference when the risk factor DM was compared between the groups of patients associated with the serum level of IL-18, showing that samples from patients with ACS, chronic CAD and healthy patients were similar to each other.

In an observational study similar to this, albeit with a prospective analysis, 194 patients, 75 of which were acute and 119 chronic, were compared with 68 controls. The analysis of smooth muscle cells from the aorta showed higher values in patients with coronary disease than in controls and that IL-18 may be an independent risk factor for CAD.^17^

In another study with 118 patients with coronary disease undergoing an angiographic study, consisting of 67 in the acute phase and 51 with stable angina, the levels of IL-18 were significantly higher in the acute ones compared to the chronic ones, which is in accordance with the results presented herein.^18^

Regarding thrombus formation, Goetze emphasizes the importance of the increase in TpP levels in patients with CAD, highlighting the relevance of having an active thrombosis marker to provide important information in the ongoing ACS.^19^

Following the same concept, Mega et al.^20^ emphasized the prognostic value of TpP in patients with ACS, showing that increased TpP levels are associated with a higher risk of death and ischemic complications, making it clear that the inclusion of an activated coagulation marker, such as TpP, in patients with established cardiovascular disease and risk factors for CAD can offer a valuable complementary analysis in the risk assessment of ACS. Carried out with 284 healthy patients and 2349 patients with ACS, this study found that the mean level of TpP was higher in patients with ACS (8.9 µg / mL vs. 3.6 µg / mL, p <0.001), which is correlated a worse prognosis.^20^ In the present study, it was observed that TpP levels were significantly higher in patients diagnosed with ACS than in patients with chronic CAD and in the control group (p <0.001). However, as this is an observational study, there was no prognostic evaluation of these patients, preventing a better analysis of the clinical outcomes that could be correlated; however, the evaluation of these data is planned.

Laurino et al.^21^ studied a cohort of 115 patients with symptoms suggestive of AMI with less than six hours duration of pain onset. Blood samples from patients were measured at 0, 1, 2, 4, 8, 16 and 24 hours after presentation for some biomarkers: total creatine kinase (total CK), CK-MB, myoglobin, troponin I and TpP. The authors observed a significant increase in serum TpP levels in 15 of the 17 patients with AMI diagnosed within six hours after symptom onset (p <0.001); 2 of the 8 patients who had AMI diagnosed six hours after symptom onset (p <0.008); 22 of the 35 patients with unstable angina (p <0.001) and 15 of the 30 patients with stable angina (p <0.001) and 3 of the 5 patients with atrial fibrillation (p <0.001); 6 of 9 patients with congestive heart failure (p <0.001); and 6 of 11 patients with non-cardiac chest pain (p <0.001). Maximum TpP concentrations in patients with AMI preceded those of the other markers by two to four hours.^21^ Therefore, acute thrombosis is associated with significant clinical alterations, including AMI. An accurate, fast and reliable test for the detection of thrombus would be an invaluable tool for doctors in the diagnosis, monitoring and treatment of patients with acute chest pain.^22^

Studies have demonstrated the importance of TpP in the assessment of the acute thrombotic process, highlighting the ACS, while others associate the increased values of this biomarker to the presence of some risk factors for CAD, such as SAH and DM in patients without evidence of ACS. As these risk factors favor a state of hypercoagulability, they may be related to a pro-thrombotic effect and may trigger an active thrombogenic process. The presence of type 2 DM may be associated with increased serum TpP values due to the predisposition of this patient profile to have markedly increased endothelial dysfunction, in addition to greater systemic coagulation activation, which caused transient alterations and favors acute cardiac events.^22 , 23 , 24^ The presence of SAH can also result in a hypercoagulable and prothrombotic environment and favor acute decompensation of coronary artery disease.^23 , 24^ The present study showed that DM and SAH did not result in a statistically significant difference between the studied groups, having not influenced the final result of the TpP measurement in relation to the different stages of coronary disease (presence of risk factor, chronic CAD and ACS). Therefore, the result obtained was not influenced by these risk factors.

### Study limitations

The sample size is small, representing a convenience sample, which can be attributed to two factors: the selection of patients carried out in hospitals that did not have an open emergency unit, making it difficult to constitute the group of acute patients. The analysis of the means found for IL-18 and TpP showed an equality between the group of chronic patients and the control group. Making a critical assessment of this observation, it can be noted that no complementary exams (e.g., carotid and vertebral Doppler) or intravascular ultrasound were performed to rule out an atherosclerotic process in other sites, which may have influenced this result, not allowing a better evaluation of biomarkers with chronicity or absence of atherosclerosis. A normal ejection fraction in all groups excluded more severe patients, which may have influenced the results. Although the present study showed that the mean serum levels of IL-18 and TpT were higher in the group of acute patients when compared to the group with stable angina or the one without coronary lesions, it is not possible to make a prognostic evaluation, as it aims to encourage the continuity of the research to confirm the hypothesis and intends to do an annual monitoring of the subjects in this sample.

## Conclusions

The mean IL-18 and TpP levels are elevated in the acute phase of coronary artery disease, suggesting the correlation of this biomarker with the worsening and the instability of acute coronary syndrome.

The mean levels of IL18 and TpP were lower in the chronic phase of coronary disease and in the patients of the Control Group; however, in this study it was observed that the Chronic Group and the Control Group had statistically equivalent means.

The results were not influenced by differences in gender or ethnicity in the groups.
